# Supplementary material for: Heat adaptation of phage T7 under an extended genetic code
Source: Virus Evol. 2021 Dec 1;7(2):veab100. doi: 10.1093/ve/veab100 (PMC8923235; doi:10.1093/ve/veab100)
Supplement: veab100_Supp [file veab100_supp.zip › Sequencing_Analysis.zip!/Sequencing_Analysis/S1/evidence/JC_SIDE_2_59.html]

BRESEQ :: Evidence


| New junction evidence | | | | | | | | | | | |
| --- | --- | --- | --- | --- | --- | --- | --- | --- | --- | --- | --- |
|  | | seq id | position | reads (cov) | reads (cov) | score | skew | freq | annotation | gene | product |
| \* | ? | NC\_001604.1 | = 16604 | 1296 (0.310) | 133 (0.030) | 92/590 | 18.3 | 8.0% | intergenic (–/–) | *–/–* | –/– |
|  | ? | NC\_001604.1 | 17028 = | 1786 (0.430) |  |  |  |  | intergenic (–/–) | *–/–* | –/– |
| Rejected: Coverage evenness skew score above cutoff. | | | | | | | | | | | |
| Rejected: Frequency below/above cutoff threshold. | | | | | | | | | | | |

Only 100 of 3897 total aligned reads displayed.  

`GACATTCTTGCGGTTGTGGTTGACGAAGATGTGCTTATTTTCACATGGGACGAAGTAAAAGGTAAGACATCCATGTGTGTCGGCAAGAGAAACAAAGGCATAAAACTATAGGAGAAATTATTATGGCTATGACAAAGAAATTTAAAGTGTCCTTCGACGTTACCGCAAAGATGTCGTCTGACGTTCAGGCAATCTTAGAGAAAGATATGCTGCATCTATGTAAGCAGGTCGGCTCAGGTGCGATTGTCCCCAATGGTAAACAGAAGGAAATGATTGTCCAGTTCCTGACACACGGTATGGAAGGATTGATGACATTCGTAGTACGTACATCATTTCGTGAGGCCATTAAGGACATGCACGAAGAGTATGCAGATAAGGACTCTTTCAAACAATCTCCTGCAACAGTACGGGAGGTGTTCTGATGTCTGACTACCTGAAAGTGCTGCAAGCAATCAAAAGTTGCCCTAAGACTTTCCAGTCCAACTATGTACGGAACAATGCGAGCCTCGTAGCGGAGGCCGCTTCCCGTGGTCACATCTCGTGCCTGACTACTAGTGGACGTAACGGTGGCGCTTGGGAAATCACTGCTTCCGGTACTCGC  >  NC_001604.1/16729‑17329`  

                                                                                                                                                                                                                                                                                                           `|`                                                                                                                                                                                                                                                                                                               
`agacaggctgggatggggactttgcgtacatggcatgggtacatgatgaaatccaaggaggatgccgtaccgaagagattgctcaggtggtcattgagaccgcacaagaagcgatgcgctgggttggagaccactggaacttccggtgtcttctggataccgaaggtaagatgggtcctaattgggcgatttgccactgatacaggaggctactcatgaacgaaagacacttaacaggtgcGATTGTCCCCAATGGTAAACAGAAGGAAATGATTGTCCAGTTCCTGACACACGGTAGGGa                                                                                                                                                                                                                                                                                                              <  1:265243‑M2/60‑1 (MQ=255)`  
  `ggataagagacaggggactttgcgtacatggcatgggtacatgatgaaatccaagtaggctgccgtaccgaagagattgctcaggtggtcattgagaccgcacaagaagcgatgcgctgggttggagaccactggaacttccggtgtcttctggataccgaaggtaagatgggtcctaattgggcgatttgccactgatacaggaggctactcatgaacgaaagacacttaacaggtgcGATTGTCCCCAATGGTAAACAGAAGGAAATGATTGTCCAGTTCCTGACACACGGTATGGAAg                                                                                                                                                                                                                                                                                                            <  1:293530‑M2/62‑1 (MQ=255)`  
  `gctggctgagaaggggactttgcgtacatagcatggggaaattatgaagtgcaagaaggctgccgtactgaagagattggtcaggtgggcattgagaccgcacaagaagcgatgcgctgggttggagaacactggaacttccggtttcttctgggtaccgaaggtaagatgggtgctaattgggcgatttgccactgatacagaaggctactaatgaacgaaagacacttaacaggtgcGATTGTCCCCAATGGTAAACAGAAGGAAATGATTGTCCAGTTCCTGACACACGGTATGGAAg                                                                                                                                                                                                                                                                                                            <  1:325933‑M2/62‑1 (MQ=255)`  
  `agagacagggatggggactttgcgtaaatggcatgggtacatgatgaaatccaagtaggctgccgtaccgaagagattgctcaggtggtcattgagaccgcacaagaagcgatgcgctgggttggagaccactggaacttccggtgtcttctggataccgaaggtaagatgggtcctaattgggcgatttgccactgatacaggaggctactcatgaacgaaagacacttaacaggtgcGATTGTCCCCAATGGTAAACAGAAGGAAATGATTGTCCAGTTCCTGACACACGGTATGGAAg                                                                                                                                                                                                                                                                                                            <  1:485764‑M2/62‑1 (MQ=255)`  
  `aatggatgggatggggactttgcgtacatggcatgggtacatgatgaaatccaagtaggctgccgtaccgaagagattgctcaggtggtcattgagaccgcacaagaagcgatgcgctgtggtggagaccactggaacttccggtgtcttctggataccgaaggtaagatgggtcctaattgggcgatttgccactgatacaggaggctactcatgaacgaaagacacttaacaggtgcGATTGTCCCCAATGGTAAACAGAAGGAAATGATTGTCCAGTTCCTGACACACGGTATGGAAg                                                                                                                                                                                                                                                                                                            <  1:548941‑M2/62‑1 (MQ=255)`  
           `gatggtgactttgcgtacatggcatgggtacatgatgaaatccaagtaggctgccgtaccgaagagattgctcaggtggtcattgagaccgcacaagaagcgatgcgctgggttggagtccactggaacttccggtgtcttctggataccgaaggtaagatgggtcctaattgggcgatttgccactgatacaggaggctactcatgaacgaaagacacttaacaggtgcGATTGTCCCCAATGGTAAACAGAAGGAAATGATTGTCCAGTTCCTGACACACGGTATGGAAGGAttgatgg                                                                                                                                                                                                                                                                                                   >  1:136366‑M2/231‑300 (MQ=255)`  
           `gatggggactttgcgtacatggcatgggtacatgatgaaatccaagtaggctgccgtaccgaagagattgctcaggtggtcattgagaccgcacaagaagcgatgcgctgggttggagaccactggaacttccggtgtcttctggataccgaaggtaagatgggtcctaattgggcgatttgccactgatacaggaggctactcatgaacgaaagacacttaacaggtgcGATTGTCCCCAATGGTAAACAGAAGGAAATGATTGTCCAGTTCCTGACacacggtatggaggctgtctctt                                                                                                                                                                                                                                                                                                   >  1:194280‑M2/231‑290 (MQ=255)`  
                  `actttgcgtacatggcatgggtacatgatgaaatccaagtaggctgccgtaccgaagagattgctcaggtggtcattgagaccgcacaagaagcgatgcgctgggttggagaccactggaacttccggtgtcttatggataccgaaggtaagatgggtcctaattgggcgatttgccactgatacaggaggctactcatgaacgaaagacacttaacaggtgcGATTGTCCCCAATGGTAAACAGAAGGACATGATTGTCCAGTTCCTGACACACGGTATGGAAGGATTGATGACATTCgt                                                                                                                                                                                                                                                                                            >  1:394778‑M2/224‑301 (MQ=255)`  
                    `tGACGAAGATGTGCTTATTTTCACATGGGACGAAGTAAAAGGTAAGACATCCATGTGTGTCGGCAAGAGAAACAAAGGCATAAAACTATAGGAGAAATTATTATGGCTATGACAAAGAAATTTAAAGTGTCCTTCGACGTTACCGCAAAGATGTCGTCTGACGTTCAGGCAATCTTAGAGAAAGATATGCTGCATCTATGTAAGCAGGTCGGCTCAGGTGCGATTGTCCCCAATGGTAAACAGAAGGAAATGATTGTCCAGTTCCTGACACACGGTATGGAAGGATTGATGACATTCgtag                                                                                                                                                                                                                                                                                          >  1:568568/1‑301 (MQ=255)`  
                      `aCGAAGATGTGCTTATTTTCACATGGGACGAAGTAAAAGGTAAGACATCCATGTGTGTCGGCAAGAGAAACACAGGCATAAAACTATAGGAGAAATTATTATGGCTATGACAAAGAAATTTAAAGTGTCCTTCGACGTTACCGCAAAGATGTCGTCTGACGTTCAGGCAATCTTAGAGAAAGATATGCTGCATCTATGTAAGCAGTTCGTCTCGGGTGCGATTGTCCCCAATGGTAACCAGAAGGAAATGATTGTCCATTTCCTGACACACGGTATGGAAAGATTGATGACATTCgtattt                                                                                                                                                                                                                                                                                        >  1:41888/1‑298 (MQ=255)`  
                         `gtaaatggcatgggtacatgatgaactccaagtaggctgccgtaccgaagagattgctcaggtggtcattgagaccgcacaagaagcgatgcgctgggttggagaccactggaacttccggtgtcttctggataccgaaggtaagatgggtcctaattgggcgatttgccactgatacaggaggctactcatgaacgaaagacacttaacaggtgcGATTGTCCCCAATGGTAAACAGAAGGAAATGATTGTCCAGTTCCTGACACACGGTATGGAAGGATTGATGACATTCGTAgtacgt                                                                                                                                                                                                                                                                                     <  1:76383‑M2/85‑1 (MQ=255)`  
                               `ggcatgggtacatgatgaaatccaagtaggctgccgtaccgaagagattgctcaggtggtcattgagaccgcacaagaagcgatgcgctgggttggagaccactggaacttccggtgtcttctggataccgaaggtaagatgggtcttaattgggcgatttgccactgatacaggaggctactcatgaacgaaagacacttaacaggtgcGATTGTCCCCAATGGTAAACAGAAGGAAATGATTGTCCAGTTCCTGACACACGGTATGGAAGGATTGATGACATTCGTAGTACGTAcatca                                                                                                                                                                                                                                                                               >  1:357314‑M2/211‑301 (MQ=255)`  
                                     `ttttCACATGGGACGAAGTAAAAGGTAAGACATCCATGTGTGTCGGCAAGAGAAACAACGGCATAAAACTATAGGAGAAATTATTATGGCTATGACAAAGAAATTTAAAGTGTCCTTCGACGTTACCGCAAAGATGTCGTCTGACGTTCAGGCAATCTTAGAGAAAGATATGCTGCATCTATGTAAGCAGGTCGGCTCAGGTGCGATTGTCCCCAATGGTAAACAGAAGGAAATGATTGTCCAGTTCCTGACACACGGTATGGAAGGATTGATGACATTCgtagctttctcttatacacat                                                                                                                                                                                                                                                                         >  1:307410/1‑284 (MQ=255)`  
                                     `ttttCACATGGGACGAAGTAAAAGGTAAGACATCCATGTGTGTCGGCAAGAGAAACAAAGGCATAAAACTATAGGAGAAATTATTATGGCTATGACAAAGAAATTTAAAGTGTCCTTCGACGTTACCGCAAAGATGTCGTCTGACGTTCAGGCAATCTTAGAGAAAGATATGCTGCATCTATGTAAGCAGGTCGGCTCAGGTGCGATTGTCCCCAATGGTAAACAGAAGGAAATGATTGTCCAGTTCCTGACACACGGTATGGAAGGATTGATGACATTCGTAGTACGTACATCATTTcct                                                                                                                                                                                                                                                                         >  1:525585/1‑299 (MQ=255)`  
                                      `gtacatgatgaaatccaagtaggctgccgtaccgaagagattgctcaggtggtcattgagaccgcacaagaagcgatgcgctgggttggagaccactggaacttccggtgtcttctggataccgaaggtaagatgggtcctaattgggcgatttgccactgatacaggaggctactcatgaacgaaagacacctaacagttgcGATTGTCCCCAATGGTAAACAGAAGGAAATGATTGTCCAGTTCCTGACACACGGTATGGAAGGATTGATGACATTCGTAGTACGTACATCATTTCGTg                                                                                                                                                                                                                                                                        >  1:70336‑M2/204‑301 (MQ=255)`  
                                           `tgatgaaatccaagtaggcttccgtaccgaagagattgctcaggtggtcattgagaccgcacaagaagcgatgcgctgggttggagaccactggaacttccggtgtcttctggataccgaaggtaagatgggtcctaattgggcgatttgccactgatacagggggctactcatgaacgaaagacacttaacaggtgcGATTGTCCCCAATGGTAAACAGAAGGAAATGATTGTCCAGTTCCTGACACACGGTATGGAAGGATTGATGACATTCGTAGTACGTACATAATTTCGTGAGGcc                                                                                                                                                                                                                                                                   >  1:344620‑M2/199‑301 (MQ=255)`  
                                            `gatgaaatccaagtaggctgccgtaccgaagagattgctcaggtggtcattgagaccgcacaagaagcgatgcgctgggttggagaccactggaacttccggtgtcttctggataccgaaggtaagatgggtcctaatttggcgatttgccactgatacaggaggctactcatgaacgaaagacacttaacaggtgcGATTGTCCCCAATGGTAAACAGAAGGAAATGATTGTCCAGTTCCTGACACACGGTATGGAAGGATTGATGACATTCGTAGTACGTACATCATTTCGTGAGGCCa                                                                                                                                                                                                                                                                  >  1:24339‑M2/198‑301 (MQ=255)`  
                                                 `aatccaagtaggctgccgtaccgaagagattgctcaggtggtcattgagaccgcacaagaagcgatgcgctgggttggagaccactggaacttccggtgtcttctggataccgaaggtaagatgggtcctaattgggcgatttgccactgatacaggaggctactcatgaacgaaagacacttaacaggtgcGATTGTCCCCAATGGTAAACAGAAGGAAATGATTGTACAGTTCCTGACACACGGTATGGAAGGATTGATGACATTCGTAGTACGTACATCATTTCGTGAGGCCATTAAg                                                                                                                                                                                                                                                             >  1:531758‑M2/193‑301 (MQ=255)`  
                                                 `aacaggTAAAAGGTAAGACATCCATGTGTGTCGGCAAGAGAAACAAAGGCATAAAACTATAGGAGAAATTATTATGGCTATGACAAAGAAATTTAAAGTGTCCTTCGACGTTACCGCAAAGATGTCGTCTGACGTTCAGGCAATCTTAGAGAAAGATATGCTGCATCTATGTAAGCAGGTCGGCTCAGGTGCGATTGTCCCCAATGGTAAACAGAAGGAAATGATTGTCCAGTTCCTGACACACGGTATGGAAGGATTGATGACATTCGTAGTACGTACATCATTTCGTGAGGCCATTAAg                                                                                                                                                                                                                                                             <  1:593992/296‑1 (MQ=255)`  
                                                   `ctcggagatgtgtaaaagagacagagattgctcaggtggtcattgagaccgaacaagaagcgatgcgctgggttggagaccactggaacttccggtgtcttctggataccgaaggtaagatgggtcctaattgggcgatttgccactgatacaggaggctactcatgaacgaaagacacttaacaggtgcGATTGTCCCCAATGGTAAACAGAAGGAAATGATTGTCCAGTTCCTGACACACGGTATGGAAGGATTGATGACATTCGTAGTACGTACATCATTTCGTGAGGCCATTAAGGa                                                                                                                                                                                                                                                           <  1:616358‑M2/111‑1 (MQ=255)`  
                                                   `aataagagacaggccgtaccgaagagattgctcaggtggtcattgagaccgaacaaggagcgatgcgctgggttggagaccactggaaattccggtgtcttctggataccgaaggtaagatgggtcctaattgggcgatttgccactgatacaggaggctactcatgaacgaaagacacttaacaggtgcGATTGTCCCCAATGGTAAACAGAAGGAAATGATTGTCCAGTTCCTGACACACGGTATGGAAGGATTGATGACATTCGTAGTACGTACATCATTTCGTGAGGCCATTAAGGa                                                                                                                                                                                                                                                           <  1:232285‑M2/111‑1 (MQ=255)`  
                                                    `aaGTAAAAGGTAAGACATCCATGTGTGTCGGCAAGAGAAACAAAGGCATAAAACTATAGGAGAAATTATTATGGCTATGACAAAGAAATTTAAAGTGTCCTTCGACGTTACCGAAAAGATGTCGTCTGACGTTCAGGCAATCTTAGAGAAAGATATGCTGCATCTATGTAAGCAGGTCGGCTCAGGTGCGATTGTCCCCAATGGTAAACAGAAGGAAATGATTGTCCAGTTCCTGACACACGGTATGGAAGGATTAATGACATTCGTAGTACGTACATCCTTTCGTgctgtctcttataca                                                                                                                                                                                                                                                          >  1:655577/1‑287 (MQ=255)`  
                                                            `gctgccgtaccgaagagattgctcaggtggtcattgagaccgcacaagaagcgatgcgctgggttggagaccactggaacttccagtgtcttctggataccgaaggtaagatgggtcctaattgggcgatttgccactgatacaggaggctactcatgaacgaaagacacttaactggtgcGATTTTCCCCAATGGTAAACAGAAGGAAATGATTGTCCAGTTCCTGACACACGGTATGTAAGGATTGATGACATTCGTAGTACGTACATCATTTCGTGAGGCCATTAAGGACATGCACGa                                                                                                                                                                                                                                                  >  1:629263‑M2/182‑301 (MQ=255)`  
                                                                `aGACATACATGTGTGTCGGCAAGAGAAACAAAGGCATAAAACTATAGGAGAAAGTATTATGGATAAGACAAAGAAATTTAAAGTGTCCTTCGACGTTACCGCAAAGATGTCGTCTGACGTTCAGGCAATCTTAGAGAAAGATATGCTGCATCTATGTAAGCAGGTCGGCTCAGGTGCGATTGTCCCCAATGGTAAACAGAAGGAAATGATTGTCCAGTTCCTGACACACGGTATGGAAGGATTGATGACATTCGTAGTACGTACATCATTTCGTGAGGCCATTAAGGACATGCACGAagag                                                                                                                                                                                                                                              <  1:382631/301‑1 (MQ=255)`  
                                                                `aGACAGCCATGTGTGTCGGCAAGAGAAACAAAGGCATAAAACTATAGGAGAAATTATTATGGCTATGACAAAGAAATTTAAAGTGTCCTTCGACGTTACCGCAAAGATGTCGTCTGACGTTCAGGCAATCTTAGAGAAAGATATGCTGCATCTATGTAAGCAGGTCGGCTCAGGTGCGATTGTCCCCAATGGTAAACAGAAGGAAATGATTGTCCAGTTCCTGACACACGGTATGGAAGGATTGATGACATTCGTAGTACGTACATCATTTCGTGAGGCCATTAAGGACATGCACGAagag                                                                                                                                                                                                                                              <  1:443771/301‑1 (MQ=255)`  
                                                                  `gtaccgaagagattgctcaggtggtcattgagaccgcacaagaagcgatgcgctgggttggagaccactggaacttccggtgtcttctggataccgaaggtaagatgggtcctaattgggcgatttgccactgatacaggaggctactcatgaacgaaagacacttaacaggtgcGATTGTCCCCAATGGTAAACAGAAGGAAATGATTGTCCAGTTCCTGACACACGGTATGGAAGGATTGATGACATTCGTCGTACGTACATCATTTCGTGAGGCCATTAAGGACATGCACGAAGAGTa                                                                                                                                                                                                                                            >  1:249081‑M2/176‑301 (MQ=255)`  
                                                                  `aCATCCATGTGTGTCGGCAAGAGAAACAAAGGCATAAAACTATAGGAGAAATTATTATGGCTATGACAAAGAAATTTAAAGTGTCCTTCGACGTTACCGCAAAGATGTCGTCTGACGTTCAGGCAATCTTAGAGAAAGATATGCTGCATCTATGTAAGCAGGTCGGCTCAGGTGCGATTGTCCCCAATGGTAAACAGAAGGAAATGATTGTCCAGTTCCTGACACACGGTATGGAAGGATTGATGACATTCGTAGTACGTACATCATTTCGTGAGGCCATTAAGGACATGCACGAAGAGtt                                                                                                                                                                                                                                            >  1:205139/1‑300 (MQ=255)`  
                                                                    `accgaagagattgctcaggtggtcattgagaccgcacaagaagcgatgcgctgggttggagaccactggaccttccggtgtcttctggataccgaaggtaagatgggtcctaattgggctatttgccactgatacaggaggctactcatgaacgaaagacacttaacaggtgcGATTGTCCCCAATGGTAAACAGAAGGAAATTATTGTCCCGTTCCTGAATCACGCTCTTGAAGGATTTATCACATTCTTAGTACGTACATCATTTCGTTAGGCTATTAAGGACATGCACGAAGAGTatt                                                                                                                                                                                                                                          >  1:376324‑M2/174‑300 (MQ=255)`  
                                                                     `tgggtataagagacaggggtgtaattgagaccgaaaaagaagcgatgcgctgggttggagaccactggaacttccggtgtcttctggataccgaaggtaagatgggtactaattgggcgatttgccactgatacaggaggctacgcatgaacgaaagacacgtaacaggtgcGATTGTCCCCAATGGTAAACAGAAGGAAATGATTGTCCAGTTACTGACACACGGTATGGAAGGATTGATGACATTCGTAGTACGTACATCATTGCGTGAGGCCATTAAGGACATGCACGAAGAGTATGc                                                                                                                                                                                                                                         <  1:364662‑M2/129‑1 (MQ=255)`  
                                                                     `cctaagagattgctcaggtggtcattgagaccgcccaagaagcgatgcgctgggttggagaccactggaacttccggtgtcttctggataccgaaggtaagatgggtcctaattgggcgatttgccactgatacaggaggctactcatgaacgaaagacacttaacaggtgcGATTGTCCCCAATGGTAAACAGAAGGAAATGATTGTCCAGTTCCTGACACACGGTATGGAAGGATTGATGACATTCGTAGTACGTACATCATTTCGTGAGGCCATTAAGGACATGCACGAAGAGTATGc                                                                                                                                                                                                                                         <  1:184281‑M2/129‑1 (MQ=255)`  
                                                                        `aTGTGTGTCGGCAAGAGAAACAAAGGCATAAAACTATATGAGAAATTATTATAGCTATGACAAAGAAATTTAAAGTGTCCTTCGACGTTACCGCAAAGATGTCGTCTGACGTTCAGGCAATCTTAGAGAAAGATATGCTGCATCTATGTAAGCAGGTCGGCTCAGGTGCGATTGTCCCCAATGGTAAACAGAAGGAAATGATTGTCCAGTTCCTGACACACGGTATGGAAGGATTGATGACATTCGTAGTACGTACATCATTTCGTGAGGCCATTAAGGACATGCACGAAGAGTATGCAGa                                                                                                                                                                                                                                      <  1:553919/301‑1 (MQ=255)`  
                                                                         `tgtgtgTCGGAAAAAGAAACAAAGGCATAAAACTATAGGAGAAATTATTATGGCTATGACAAAGAAATGTAAAGTGTCCTTCGACGTTACCGAAAAGATGGCGTCTGACGTTCAGGCAATCTTAGAGAAAGATATGCTGCATCTATGTAAGCAGGTCGGCTCAGGTGCGATTGTCCCCAATGGTAAACAGAAGGAAATGATTGTCCAGTTCCTGACACACGGTATGGAAGGATTGATGACATTCGTAGTACGTACATCATTTCGTGAGGCCATTAAGGACATGCACGAAGAGTATGCAGAt                                                                                                                                                                                                                                     <  1:81200/301‑1 (MQ=255)`  
                                                                           `agattgctcaggtggtcattgagaccgcacaagaagcgatgcgctgggttggagaccactggaacttccggtgtcttctggataccgaaggtaagatgggtcctaattgggcgatttgccactgatacaggaggctactcatgaacgaaagacacttaacaggttcGATTGTCCCCAATGGTAAACAGAAGGAAATGATTGTCCAGTTCCTGACACACGGTATGGAAGGATTGATGACATTCGTAGTACGTACATCATTTCGTGAGGCCATTAAGGACATGCACGAAGAGTATGCAGATaa                                                                                                                                                                                                                                   >  1:667620‑M2/167‑301 (MQ=255)`  
                                                                            `gtgtCGGCAAGAGAAACAAAGGCATAAAACTATAGGAGAAATTATTATGGCTATGACAAAGAAATTTAAAGTGTCCTTCGACGTTACCGCAAAGATGTCGTCTGACGTTCAGGCAATCTTAGAGAAAGATATGCTGCATCTATGTAAGCAGGTCGGCTCAGGTGCGATTGTCCCCAATGGTAAACAGAAGGAAATGATTGTCCAGTTCCTGACACACGGTATGGAAGGATTGATGACATTCGTAGTACGTACATCATTTCGTGAGGCCATTAAGGACATGCACGAAGAGTATGAAgctgtt                                                                                                                                                                                                                                  >  1:111809/1‑296 (MQ=255)`  
                                                                               `tgctcaggtggtcattgggacagcacaagaagcgatgcgctgggtttgagaccactggaacttccggtgtcttctggttacccaaggtaagatgggtcctaattgggcgatttgccactgatacaggaggctactcatgaacgaaagacacttaacaggtacGATTGTCCCCAATAGTAAACAGAAGGAAATGATTGTCCAGTTCCTGACACACGGTATGGAAGGATTGATGACATTCGTAGTACGTACATCATTTCGTGAGGCCATTAAGGACATGCACGAAGAGTATGCAGATAAGGAc                                                                                                                                                                                                                               <  1:159906‑M2/139‑1 (MQ=255)`  
                                                                               `tataagagacagcatggagaccgcacaagaagcgattcgctgggttggagaccactggaacttccggtgtcttctggataccgaaggtaagatgggtcctaattgggcgatttgccactgatacaggaggctacttatgaacgaaagacacttaacaggtgcGATTGTCCCCAATGGTAAACAGAAGGAAATGATTGTCCAGTTCCTGACACACGGTATGGAAGGATTGATGACATTCGTAGTACGTACATCATTTCGTGAGGCCATTAAGGACATGCACGAAGAGTATGCAGATAAGGAc                                                                                                                                                                                                                               <  1:515762‑M2/139‑1 (MQ=255)`  
                                                                               `agagacagtggtcattgagaccgaacaagaagcgatgcgctgggttggagaccactggaacttccggtgtcttctggataccgaaggtaagatgggtcctaattgggcgatttgccactgatacaggaggctactcatgaacgaaagacacttaacaggtgcGATTGTCACAAATGGTAAACAGAAGGAAATGATTGTCCAGTTCCTGACACACGGTATGGAAGGATTGATGACATTCGTAGTACGTACATCATTTCGTGAGGCCATTAAGGACATGCACGAAGAGTATGCAGATAAGGAc                                                                                                                                                                                                                               <  1:370189‑M2/139‑1 (MQ=255)`  
                                                                                `gctcaggtggtcattgagaccgcacaagaagcgatgcgctgggttggagaccactggaacttccggtgtcttctggataccgaaggtaagatgggtcctaattgggcgatttgccactgatacaggaggctactcatgaacgaaagacacttaacaggtgcGATTGTCCCCAATGGTAAACAGAAGGAAATGATTGTCCAGTTCCTGACACACGGTATGGAAGGATTGATGACATTCGTAGTACGTACATCATTTCGTGAGGCCATTAAGGACATGCACGAAGAGTATGCAGATAAGGacc                                                                                                                                                                                                                              >  1:497193‑M2/162‑300 (MQ=255)`  
                                                                                     `ggtggtcattgagaccgcacaagaagcgatgcgctgggttggagaccactggaacttccggtgtcttctggataccgaaggtaagatgggtcctaattgggcgatttgccactgatacaggaggctactcatgaacgaaagacacttaacaggtgcGATTGTCCCCAATGGTAAACAGAAGGAAATGATTGTCCAGTTCCTGACACACGGTATGGAAGGATTGATGACATTCGTAGTACGTACATCATTTCGTGAggccattaaggacatgcacgaagagctgtctcttatccacatctcc                                                                                                                                                                                                                         >  1:468834‑M2/157‑280 (MQ=255)`  
                                                                                         `aaaCAAAGGCATAAAACTATAGGAGAAATTATTATGGCTATGACAAAGAAATTTAAAGTGTCCTTCGACGTTACCGCAAAGATGTCGTCTGACGTTCAGGCAATCTTAGAGAAAGATATGCTGCATCTATGTAAGCAGGTCGGCTCAGGTGCGATTGTCCCCAATGGTAAACAGAAGGAAATGATTGTCCAGTTCCTGACACACGGTATGGAATGATTGATGACATTCGTAGTACGTACATCATTTCGTGAGGCCATTAAGGACATGCACGAAGAGTATGCAGATAAGGACTCTTTCAAAc                                                                                                                                                                                                                     <  1:129355/301‑1 (MQ=255)`  
                                                                                           `cattgagaccgcacaagaagcgatgcgctgggttggagaccactggaacttccggtgtcttctggataccgaaggtaagatgggtcctaattgggcgatttgccactgatacaggaggctactcatgaacgaaagacgcttaacaggtgcGATTGTCCCCAATGGTAAACAGAAGGAAATGATTGTCCAGTTCCTGACACACGGTATGGAAGGATTGATGACATTCGTAGTACGTACATCATTTCGTGAGGCCATTAAGGACATGCACGAAGAGTAtgcagataaggacctgtctcgtata                                                                                                                                                                                                                   >  1:280020‑M2/151‑289 (MQ=255)`  
                                                                                           `cattgagaccgcacaagaagcgatgcgctgggttggagaccactggaacttccggtgtcttctggataccgaaggtaagatgggtcctaattgggcgatttgccactgatacaggaggctactcatgaacgaaagacacttaacaggtgcGATTGTCCCCAATGGTAAACAGAAGGAAATGATTGTCCAGTTCCTGACACACGGTATGGAAGGATTGATGACATTCGTAGTACGTACATCATTTCGTGAGGCCATTAAGGACATGCACGAAGAGTATGCAGATAAGGACTCTTTCAAACaa                                                                                                                                                                                                                   >  1:154585‑M2/151‑301 (MQ=255)`  
                                                                                                      `cacaagaagcgatgcgctgggttggagaccactggaacttccggtgtctgctggataccgaaggtaagatgggtcctaattgggcgatttgccactgatacaggaggctactcatgaacgaaagacacttaacaggtgcGATTGTCCCCAATGGTAAACAGAAGGAAATGATTGTCCAGTTCCTGACACACGGTATGGAAGGATTGATGACATTCGTAGTACGTACATCATTTCGTGAGGCCATTAAGGACATGCATGAAGAGTATGCAGATAAGGACTCTTTCAAACAATCTCCTGCAAc                                                                                                                                                                                                        <  1:462314‑M2/162‑1 (MQ=255)`  
                                                                                                       `aaCTATAGGAGAAATTATTATGGCTATGACAAAGAAATTTAAAGTGTCCTTCGACGTTACCGAAAAGATGTCGTCTGACGTTCAGGCAATCTTAGAGAAAGATATGCTGCATCTATGTAAGCAGGTCGGCTCAGGTGCGATTGTCCCCAATGGTAAACAGAAGGAAATGATTGTCCAGTTCCTGACACACGGTATGGAAGGATTGATGACATTCGTAGTACGTACATCATTTCGTGAGGCCATTAAGGACATGCACGAAGAGTATGCAGATAAGGACTCTTTCAAACAATCTCCTGCAACa                                                                                                                                                                                                       >  1:189378/1‑301 (MQ=255)`  
                                                                                                              `ggAGAAATTATTATGGCTATGACAAAGAAATTTAAAGTGTCCTTCGACGTTACCGCAAAGATGTCGTCTGACGTTCAGGCAATCTTAGAGAAAGATATGCTGCATCTATGTAAGCAGGTCGGCTCAGGTGCGATTGTCCCCAATGGTAAACAGAAGGAAATGATTGTCCAGTTCCTGACACACGGTATGGAAGGATTGATGACATTCGTAGTACGTACATCATTTCGTGAGGCCATTAAGGACATGCACGAAGAGTATGCAGATAAGGACTCTTTCAAACAATCTCCTGCAACAGTACggg                                                                                                                                                                                                >  1:301969/1‑301 (MQ=255)`  
                                                                                                                      `tattatGGCTATGACAAAGAAATTTAAAGTGTCCTTCGACGTTACCGCAAAGATGTCGTCTGACGTTCAGGCAATCTTAGAGAAAGATATGCTGCATCTATGTAAGCAGGTCGGCTCAGGTGCGATTGTCCCCAATGGTAAACAGAAGGAAATGATTGTCCAGTTCCTGACACACGGTATGGAAGGATTGATGACATTCGTAGTACGTACATCATTTCGTGAGGCCATTAGGGACATGCACGAAGAGTATGCAGATAAGGACTCTTTCAAACAATCTCCTGCAACAGTACGGGAGGTGTTc                                                                                                                                                                                        >  1:338617/1‑301 (MQ=255)`  
                                                                                                                        `gggttggagaccactggaacttccggtgtcttctggataccgaaggtaagatgggtcctaattgggcgatttgccactgatacaggaggctactcatgaacgaaagacacttaacaggtgcGATTGTCCCCAATGGTAAACAGAAGGAAATGATTGTCCAGTTCCTGACACACGGTATGGAAGGATTGATGACATTCGTAGTACGTACATCATTTCGTGAGGCCATTAAGGACATGCACGAAGAGTATGCAGATAAGGACTATTTCAAACAATCTCCTGCAACAGTACGGGAGGTGTTctt                                                                                                                                                                                      >  1:218443‑M2/122‑300 (MQ=255)`  
                                                                                                                        `gggttggagaccactggaacttccggtgtcttctggataccgaaggtaagatgggtcctaattgggcgatttgccactgatacaggaggctactcatgaacgaaagacacttaacaggtgcGATTGTCCACAATGGTAAACAGAAGGAAATGATTGTCCAGTTCCTGACACACGGTATGGAAGGATTGATGACATTCGTAGTACGTACATCATTTCGTGAGGCCATTAAGTACATGCACGAAGAGTATGCAGATAAGGACTCTTTCAAACAATCTCCTGCAACAGTACGGGAGGTGTTCTg                                                                                                                                                                                      >  1:165518‑M2/122‑301 (MQ=255)`  
                                                                                                                         `tatGGCTATGACAAAGAAATTTAAAGTGTCCTTCGACGTTACCGCAAAGATGTCGGCTGACGTTCAGGCAATCTTATAGAAAGATATGCTGCATCTATGTAAGCAGGTCGGCTCAGGTGCGATTGTCCCCAATGGTAAACAGAAGGAAATGATTGTCCAGTTCCTGACACACGGTATGGAAGGATTGATGACATTCGTAGTACGTACATCATTTCGTGAGGCCATTAAGGACATGCACGAAGAGTATGCAGATAAGGACTCTTTCAAACAATCTCCTGCAACAGTACGGGAGGTGTTCTGa                                                                                                                                                                                     <  1:122872/301‑1 (MQ=255)`  
                                                                                                                         `atgtgtataagagacagaaTTTAAAGTGTCCTTCGACGTTACCGCAAAGATGTCGTCTGACGTTCAGGCAATCTTAGAGAAAGATATGCTGCATCTATGTAAGCAGGTCGGCTCAGGTGCGATTGTCCCCAATGGTAAACAGAAGGAAATGATTGTCCAGTTCCTGACACACGGTATGGAAGGATTGATGACATTCGTAGTACGTACATCATTTCGTGAGGCCATTAAGGACATGCACGAAGAGTATGCAGATAAGGACTCTTTCAAACAATCTCCTGCAACAGTACGGGAGGTGTTCTGa                                                                                                                                                                                     <  1:86650/284‑1 (MQ=255)`  
                                                                                                                          `gttggagaccactggaacttccggtgtcttctggataccgaaggtaagatgggtcctaattgggcgatttgccactgatacaggaggctactcatgaacgaaagaaacttaacaggtgcGATTGTCCCCAATGGTAAACAGAAGGACATGATTGTCCAGTTCCTGACACACGGTATGGAAGGATTGATGACATTCGTAGTACGTACATCATTTCGTGAGGCCATTAAGGAAATGCACGAAGAGTATGCAGATAAGGACTCTTTATAAACATCTCCTGCAACAGTACGGGaggagtttagat                                                                                                                                                                                    >  1:389213‑M2/120‑296 (MQ=255)`  
                                                                                                                                 `tGACAAAGAAATTTAAAGTGTCCTTCGACGTTACCGCAAAGATGTCGTCTGACGTTCAGGCAATCTTAGAGAAAGATATGCTGCATCTATGTAAGCAGGTCGGCTCAGGTGCGATTGTCCCCAATGGTAAACAGAAGGAAATGATTGTCCAGTTCCTGACACACGGTATGGAAGGATTGATGACATTCGTAGTACGTACATCATTTCGTGAGGCCATTAAGGACATGCACGAAGAGTATGCAGATAAGGACTCTTTCAAACAATCTCCTGCAACAGTACgggctgtctcttatacacatct                                                                                                                                                                             >  1:537837/1‑282 (MQ=255)`  
                                                                                                                                  `ccactggaacttccggtgtcttctggataccgaaggtaagatgggtcctaattgggcgatttgccactgatacaggaggctactcatgaacgaaagacacttaacaggtgcGATTGTCCCCAATGGTAAACAGAAGGAAATGATTGTCCAGTTCCTGACACACGGTATGGAAGGATTGATGACATTCGTAGTACGTATATCATTTCGTGAGGCCATTAAGGACATGCACGAAGAGTATGCAGATAAGGACTCTTTCAAACAATCTCCTGCAACAGTACGGGAGGTGTTCTGATGTCTGACt                                                                                                                                                                            >  1:117987‑M2/112‑301 (MQ=255)`  
                                                                                                                                                `ggtgtcttctggataccgaaggtaagatgggtcctaattgggcgatttgccactgatacaggaggctactcatgaacgaaagacacttaacaggtgcGATTGTCCCCAATGGTAAACAGAAGGAAATGATTGTCCAGTTCCTGACACACGGTATGGAAGGATTGATGACATTCGTAGTACGTACATCATTTCGTGAGGCCATTAAGGACATGCACGAAGAGTATGCAGATAAGGCCTCTTTCAAACAATCTCCTGCAACAGTACGGGAGGTGTTATGATGTCTGACTACCTGAAAGtgctg                                                                                                                                                              >  1:425709‑M2/98‑301 (MQ=255)`  
                                                                                                                                                   `gtcttctggataccgaaggtaagatgggtcctaattggtcgatttgccactgatacaggaggctactcatgaacgaaagacacttaacaggtgcGATTGTCCCCAATGGTAAACAGAAGGAAATGATTGTCCAGTTCCTGACACACGGTATGGAAGGATTGATGACATTCGTAGTACGTACATCATTTCGTGAGGCCATTAAGGACATGAACGAAGAGTATGCAGATAAGGACTCTTTCAAACAATCTCCTGCAACAGTACGGGAGGTGTTCTGATGTCTGACTACCTGAAAGTGCTgcaa                                                                                                                                                           >  1:272481‑M2/95‑301 (MQ=255)`  
                                                                                                                                                                 `aCCGCAAAGATGTCGTCTGACGTTCAGGCAATCTTAGAGAAAGATATGCTGCATCTATGTAAGCAGGTCGGCTCAGGTGCGATTGTCCCCAATGGTAAACAGAAGGAAATGATTGTCCAGTTCCTGACACACGGTATGGAAGGATTGATGACATTCGTAGTACGTACATCATTTCGTGAGGCCATTAAGGACATGCACGAAGAGTATGCAGATAAGGACTCTTTCAAACAATCTCCTGCAACAGTACGGGAGGTGTTCTGATGTCTGACTACCTGAAAGTGCTgcaagcctgtctcttatt                                                                                                                                             >  1:575227/1‑289 (MQ=255)`  
                                                                                                                                                                          `atgggtcctgattgggagatatggcactgatacaggaggatactcatgaacgaaagacacttaacaggtgcGATTGTCCCCAATGGTAAACAGAAGGAAATGATTGTCCAGTTCCTGACACACGGTATGGAAGGATTGATGACATTCGTAGTACGTACATCATTTCGTGAGGCCATTAAGGACATGCACGAAGAGTATGCAGATAAGGACTCTTTCAAACAATCTCCTGCAACAGTACGGGAGGTGTTCTGATGTCTGACTACCTGAAAGTGCTGCAAGCAATCAAAAGTTGCCCTAAGAc                                                                                                                                    <  1:254409‑M2/230‑1 (MQ=255)`  
                                                                                                                                                                            `gtcgtcTGACGTTCAGGCAATCTTAGAGAAAGATATGCTGCATCTATGTAAGCAGGTCGGCTCAGGTGCGATTGTCCCCAATGGTAAACAGAAGGAAATGATTGTCCAGTTCCTGACACACGGTATGGAAGGATTGATGACATTCGTAGTACGTACATCATTTCGTGAGGCCATTAAGGACATGCACGAAGAGTATGCAGATAAGGACTCTTTTAAACAATCTCCGGCAACAGTACGGGAGGTGTTCTGATGTCTTACTACCTGAAATTGCTGCAAGCAATCAAAAGTTGCCCTAAGACtt                                                                                                                                  >  1:563981/1‑301 (MQ=255)`  
                                                                                                                                                                             `ggtcctaattgggcgatttgccactgatacaggaggctactcatgaacgaaagacacttaacaggtgcGATTGTCCCCAATCGTAAACAGAAGGAAATGATTTTCCAGTTCCTGACACACGGTATGGAAGGATTGATGACATTCGTAGTACGTACATCATTTCGTGAGGCCATTAAGGACATGCACGAAGATTATGCAGATAAGGACTCTTTCAAACAATCTCCTGCAACAGTACGGGCAGTGTTCTGATGTCTGACTACCTGACAGTGCTGCAAGCAATCAAAAGTTTCCCTAAGACttt                                                                                                                                 >  1:635934‑M2/69‑301 (MQ=255)`  
                                                                                                                                                                              `gtcctaattgggcgatttgccactgatacaggaggctactcatgaacgaaagacacttaacaggtgcGATTGTCCCCACTGGTAAACAGAAGGAAATGATTGTCCAGTTCCTGACACACGGTATGGCAGGATTGATGACATTCGTAGTACGTACATCATTTCGTGAGGCCATTAAGGACATGCACGCAGAGTATGCAGATAAGGACTCTTTCAAACAATCTCCTGCAACAGTGCGGGAGGTGTTCTGATGTCTGACTACCTGAACGTGCTGCAAGCAATCAACAGTTGCCCTAAGAATTTc                                                                                                                                >  1:600060‑M2/68‑301 (MQ=255)`  
                                                                                                                                                                               `tcctaattgggcgatttgccactgatacaggaggctactcatgaacgaaagacacttaacaggtgcTCTTGTCCCCAATGGTAAACAGACGGAAATGATTGTCCAGTTCCTGACACACGGTATTGAAGGATTGATGACATTCTTATTACGTACATCATTTCGTGAGGCCATTAAGGACATGCACGAAGCGTATGCAGATAAGGACTCTTTCAAACAATCTCCTGCAACAGTACGGGAGGTGTTCTGATGTCTGACTACCTGAAAGTGCTGCAAGCAATCACAAGTTGCCCTAAGACTTTcc                                                                                                                               >  1:660906‑M2/67‑301 (MQ=255)`  
                                                                                                                                                                                 `ctaattgggcgatttgccactgatacaggaggctactcatgaacgaaagacacttaacaggtgcGATTGTCCCCAATGGTAAACAGAAGGAAATGATTGTCCAGTTCCTGACACACGGTATGGAAGGATTGATGACATTCGTAGTACGTACATCATTTCGTGAGGCCATTAAGGACATGCACGAAGAGTATGCAGATAATGACTCTTTCACACAATCTCCTGCAACAGTACGGGAGGTGTTCTGATGTCTTACTATCTGAAAGTGCTGCAAGCAATCAAAAGTTGCCCTAAGACGttccct                                                                                                                             >  1:431506‑M2/65‑299 (MQ=255)`  
                                                                                                                                                                                   `gACGTTCAGGCAATCTTAGTGAAAGATATGCTGCATCTATGTAAGCAGGTCGGCTCAGGTGCGATTGTCCCCAATGGTAAACAGAAGGAAATGATTGTCCAGTTCCTGACACACGGGATGGAAGGATTGATGACATTCGTAGTACGTACATCATTTCGTGAGGCCATTAAGGACATGCACGAAGAGTATGCAGATAAGGACTCTTTCAAACAATCTCCTGCAACAGTACGGGAGGTGTTCTGATGTCTGACTACCTGAAAGTGCTGAAAGCAATCAAAAGTTTCCCTAAGCATTTCCAGTc                                                                                                                           >  1:558261/1‑301 (MQ=255)`  
                                                                                                                                                                                               `aTCTTAGAGAAAGATATGCTGCATCTATGTAAGCAGGTCGGCTCAGGTGCGATTGTCCCCAATGGTAAACAGAAGGAAATGATTGTCCAGTTCCTGACACACGGTATGGAAGGATTGATGACATTCGTAGTACGTACATCATTTCGTGAGGCCATTAAGGACATGCACGAAGAGTATGCAGATAAGGACTCTTTCAAACAATCTCCTGCAACAGTACGGGAGGTGTTCTGATGTCTGACTACCTGAAAGTGCTGCAAGCAATCAAAAGTTGCCCTAAGACTTTCCAGTCCAACTATgcggt                                                                                                               >  1:642025/1‑297 (MQ=255)`  
                                                                                                                                                                                               `aTCTTAGAGAAAGATATGCTGCATCTATGTAAGCAGGTCGGCTCAGGTGCGATTGTCCCCAATGGTAAACAGAAGGAAATGATTGTCCAGTTCCTGACACACGGTATGGAAGGATTGATGACATTCGTAGTACGTACATCATTTCGTGAGGCCATTAAGGACATGCACGAAGAGTATGCAGATAAGGACTCTTTCAAACAATCTCCTGCAACAGTACGGGAGGTGTTCTGATGTCTGACTACCTGAAAGTGCTGCAAGCAATCAAAAGTTGCCCTAAGACTTTCCAGTCCAACTATGTACg                                                                                                               <  1:198627/301‑1 (MQ=255)`  
                                                                                                                                                                                                 `ggggatgtggataaggggcagcatgaacgaaatacacttaacaggtgcGATTGTCCCCAATGGTAAACAGAAGGAAATGATTGTCCAGTTCCTGACACACGGTATGGAAGGATTGATGACATTCGTAGTACGTACATCATTTCGTGAGGCCATTAAGGACATGCACGAAGAGTATGCAGATAAGGACTCTTTCAAACAATCTCCTGCAACAGTACGGGAGGTGTTCTGATGTCTGACTACCTGAAAGTGCTGCAAGCAATCAAAAGTTGCCCTAAGACTTTCCAGTCCAACTATGTACGGa                                                                                                             <  1:401416‑M2/253‑1 (MQ=255)`  
                                                                                                                                                                                                   `tAGAGATATATTTGCTTCATCTATGTAAGCAGGTCGTCTCAGGTGCCATTGTCCCCAATGGTAAACAGAATGAAATGATTGTTCAGTTCCTGACACACTGTATGTCAGGATTGATGACATTCGTAGTACGTACATCATTTCGTGATGCCATTAAGGACATGCACGAAGATTATGCAGATAAGGACTCTTTCAAACAATCTCCTGCAACAGTACGGGAGGTGTTCTGATGTCTGACTACCTGAAAGTGCTGCAAGCAATCAAAAGTTGCCCTAAGACTTTCCAGTCCAACTATGTACGGAAc                                                                                                           <  1:587099/301‑1 (MQ=255)`  
                                                                                                                                                                                                   `ggagatgtgtataagagacagtgaacgaaagacacttaacaggtgcGATTGTCCCCAATGGTAAACAGAAGGAAATGATTGTCCAGTTCCTGACACACGGTATGGAAGGATTGATGACATTCGTAGTACGTACATCATTTCGTGAGGCCATTAAGGACATGCACGAAGAGTATGCAGATAAGGACTCTTTCAAACAATCTCCTGCAACAGTACTGGAGGTGTTCTGATGTCTGACTACCTGAAAGTGCTGCAAGCAATCAAAAGTTGCCCTAAGACTTTCCAGTCCAACTATGTACGGAAc                                                                                                           <  1:237121‑M2/255‑1 (MQ=255)`  
                                                                                                                                                                                                     `tgatacaggaggctactcatgaacgaaagacacttaacaggtgcGATTGTCCCCAATGGTAAACAGAAGGAAATGATTGTCCAGTTCCTGACACACGGTATGGAAGGATTGATGACATTCGTAGTACGTACATCATTTCGTGAGGCCATTAAGGACATGCACGAAGAGTATGCAGATAAGGACTCTTTCAAACAATCTCCTGCAACAGTACGGGAGGTGTTCTGATGTCTGACTACCTGAAAGTGCTGCAAGCAATCAAAAGTTGCCCTAAGactttccagtccaacctgtctcttataca                                                                                                         >  1:212759‑M2/45‑287 (MQ=255)`  
                                                                                                                                                                                                      `gatacaggaggctactcatgaacgaaagacacttaacaggtgcGATTGTCCCCAATGGTAAACAGAAGGAAATGATTGTCCAGTTCCTGACACACGGTATGGAAGGATTGATGACATTCGTAGTACGTACATCATTTCGTGAGGCCATTAAGGACATGCACGACGAGTATGCAGATAAGGACTCTTTCAAACAATCTCCTGCAACAGTACGGGGGGTGTTCTGATGTCTGACTACCTGAAAGTGCTGCAAGCAATCAAAAGTTGCCCTACGACTTTCCAGCCCAACTATGTCAGGAACAAt                                                                                                        >  1:172730‑M2/44‑301 (MQ=255)`  
                                                                                                                                                                                                       `atacaggaggctactcatgaacgaaagacacttaacaggtgcGATTGTCCCCAATGGTAAACAGAAGGAAATGATTGTCCAGTTCCTGACACACGGTATGGAAGGATTGATGACATTCGTAGTACGTACATCATTTCGTGAGGCCATTAAGGAAATGCACGAAGAGTATGCCGATAAGGACTCTTTCAAACAAACTCCTGCAACAGTACGGGTAGTGTTCTGAAGTCTGACTACCTGAAAGTGCTGCAAGCAAACAAAAGTTGCCCTAAGACTTTCCAGTCCaactatgtacatgtctctt                                                                                                       >  1:480774‑M2/43‑292 (MQ=255)`  
                                                                                                                                                                                                          `aGATATGCTGCATCTATGTAAGCAGGTCGGCTCAGGTGCGATTGTACCCAATGGTAAACAGAAGGAAATGATTGTCCAGTTCCTGACACACGGTATGGAAGGATTGATAACATTAGAAGTACGTACATCATTACGTGAGGCCATTAAGGCCATGCACGAAGAGTATGAAGATAAGGACTCTTTCAAACAATCGCCAGCAACAGAAAGGGAGGTGTTCTGATGTCTGACTACCTGAAAGTGATGCAAGCAATAAAAAGTGTCCCTAAAACTTTCCGATCCAACTATGCAGGGAaaaaggagg                                                                                                    >  1:411532/1‑296 (MQ=255)`  
                                                                                                                                                                                                           `gATATGCTGCATCTATGTAAGCAGGTCGGCTCAGGTGCGATTGTCCCCAATGGTAAACAGAAGGAAATGATTGTCCAGTTCCTGACACACGGTATGGAAGGATTGATGACATTCGTAGTACGTACATCATTTCGTGAGGCCATTAAGGACATGCACGAAGAGTATGCAGATAAGGACTCTTTCAAACAATCTCCTGCAACAGTACGGGAGGTGTTCTGATGTCTGACTACCTGAAAGTGCTGCAAGCAATCAAAAGTTGTCCTAAGACTTTACAGTCCAACTATGTACGGAACAATGCGAg                                                                                                   >  1:224781/1‑301 (MQ=255)`  
                                                                                                                                                                                                            `ggaggctactcatgaacgaaagacacttaacaggtgcGATTGTCCCCAATGGTAAACAGAAGGAAATGATTGTCCAGTTCCTGACACACGGTATGGAAGGATTGATGACATTCGTAGTACGTACATCATTTCGTGAGGCCATTAAGGACATGCACGAAGAGTATGCAGATAAGGACTCTTTCAAACAATCTCCTGCAACAGTACGGGAGGTGTTCTGATGTCTGACTACCTGAAAGTGCTGCAAGCAATCAAAAGTTGCCCTaagactttccagtccaactactgtctcttatacacatct                                                                                                  >  1:491330‑M2/38‑282 (MQ=255)`  
                                                                                                                                                                                                                 `ctactcatgaacgaaagacacttaacaggtgcGATTGTCCCCAATGGTAAACAGAAGGAAATGATTGTCCAGTTCCTGACACACGGTATGGAAGGATTGATGACATTCGTAGTACGTACATCATTTCGTGAGGCCATTAAGGACATGCACGAAGAGTATGCAGATAAGGACTCTTTCAAACAATCTCCTGCAACAGTACGGGAGGTGTTCTGATGTCTGACTACCTGAAAGTGCTGCAAGCAATCAAAAGTTGACCTAAGACTTTCCAGTCCAACTATGTACGGAACAATGCGCGCCTCGt                                                                                             >  1:258933‑M2/33‑301 (MQ=255)`  
                                                                                                                                                                                                                    `ctcatgaacgaaagacacttaacaggtgcGATTGTCCCCAATGGTAAACAGAAGGAAATGATTGTCCAGTTCCTGACACACGGTATGGAAGGATTGATGACATTCGTAGTACGTACATCATTTCGTGAGGCCATTAAGGACATGCACGAAGAGTATGCAGATAAGGACTCTTTCAAACAATCTCCTGCAACAGTACGGGAGGTGTTCTGATGTCTGACTACCTGAAAGTGCTGCAAGCAATCAAAAGTTGCCCTAAGACTTTCCAGTCCAACTATGTACGGAACAATGCGAGCCTCGTagg                                                                                          >  1:543751‑M2/30‑300 (MQ=255)`  
                                                                                                                                                                                                                      `cattaacgaaagacacttaacaactgcGATGGTCCCCAATGGTAAACAGAAGTAAATGATTGTCCAGTTCCTGACACACTGTATGGAAGGATTTATGACATTCGTAGTACGTACATCATTTCGTGAGGCCATTAAGGACATGCACGAAGAGTATTCATATAAGTACTCTTTCAACCAATCTCCTGCAACAGTACGGGATGTGTTCTGATGTCTGACTACCTGAAAGTGCTGCAAGCAATCAAAAGTTGCCCTAAGACTTTCCAGTCCAACTATGTACGGAACAATGCGAGCCTCGTAGCgg                                                                                        <  1:405012‑M2/274‑1 (MQ=255)`  
                                                                                                                                                                                                                         `aTGTAAGCAGGTCGGCTCAGGTGCGATTGTCCCCAATGGTAAACAGAAGGAAATGATTGTCCAGTTCCTGACACACGGTATGGAAGGATTGATGACATTCGTAGTACGTACATCATTTCGTGAGGCCATTAAGGACATGCACGAAGAGTATGCAGATAAGGACTCTTTCAAACAATCTCCTGCAACAGTACGGGAGGTGTTCTGATGTCTGACTACCTGAAAGTGCTGCAAGCAATCAAAAGTTGCCCTAAGACTTTCCAGTCCAACTATGTACGGAACAATGCGAGCCTCGTAGCGGAgg                                                                                     <  1:606351/301‑1 (MQ=255)`  
                                                                                                                                                                                                                         `aTGTAAGCAGGTCGGCTCAGGTGCGATTGTCCCCAATGGTAAACAGAAGGAAATGATTGTCCAGTTCCTGACACACGGTATGGAAGGATAGATGACATTCGTAGTACGTACATCATTTCGTGAGGCCATTAAGGACATGCACGAAGAGTATGCAGATAAGGACTCTTTCAAACAATCTCCTGAAACAGTACGGGAGGTGTTCTGATGTCTGACTACCTGAAAGTGCTGCAAGAAATCAAAAGTTGCCCTAAGACTTTCCAGTCAAACTATGTACGGAACAATGCGACTCTCGTAGCGGAgg                                                                                     >  1:623163/1‑301 (MQ=255)`  
                                                                                                                                                                                                                                  `ggTCGGCTCAGGTGCGATTGTCCCCAATGGTAAACAGAAGGAAATGATTGTCCAGTTCCTGACACACGGTATGGAAGGATTGATGACATTCGTAGTACGTACATCATTTCGTGAGGCCATTAAGGACATGCACGAAGAGTATGCAGATAAGGACTCTTTCAAACAATCTCCTGCAACAGTACGGGAGGTGTTCTGAGGTCTGACTACCTGAAAGTGCTGCAAGCAATCAAAAGTTGCCCTAAGACTTTCCAGTCCAACTATGTACGGAACAATTCGAGCCTCGTAGCGGAGGCCGCTTccc                                                                            >  1:454917/1‑301 (MQ=255)`  
                                                                                                                                                                                                                                     `cttaacaggtgcGATTGTCCCCAATGGTAAACAGAAGGAAATGATTGTCCAGTTCCTGACACACGGTATGGCAGGATTGATGACATTCGTAGTACGTACATCATTTCGTGAGGCCATTAAGGACATGCACGAAGAGTATGCAGATCAGGACTCTTTCACACAATCTCCTGCAACAGTACGGGAGGTTTTCTGATGTCTGACTACCTGAAAGTGCTGCAAGCAATCAAAAGTTGCCCTAAGACTTTCCATTCCAACTATGTACGGAACAATGCGAGCCTCGTAGCGGAGGCAGCGTACCGTg                                                                         >  1:351171‑M2/13‑301 (MQ=255)`  
                                                                                                                                                                                                                                                       `ccccAATGGTAAACAGAAGGAAATGATTGTCCAGTTCCTGACACACGGTATGGAAGGATTGATGACATTCGTAGTACGTACATCATTTCGTGATGCCATTAAGGACATGCACGAAGAGTATGCAGATAAGGACTCTTTCAAACAATCTCCTGCAACAGTACGGGAGGTGTTCTGATGTCTGACTACCTGAAAGTGCTGCAAGCAATCAAAAGTTGCCCTAAGACTTTCCAGTCCAACTATGTACGGAACAATGCGAGCCTCGTAGCGGAGGCCGCTTCCCGTGGTCACATCTCGTTCCTGa                                                       >  1:449414/1‑301 (MQ=255)`  
                                                                                                                                                                                                                                                            `aTGGTAAACAGAAGGAAATGATTGTCCAGTTCCTAACACACGGTATGGAAGGATTGATGACATTCGTAGTACGTACATCATTTCGTGAGGCCATTAAGGACATGCACGAAGAGTATGCAGATAAGGACTCTTTCAAACAATCTCCTGCAACAGTACGGGAGGGGTACTGATGTCTGACTACCTGAAAGTGCTGCAAGCAAACAAACGTTGCCCTAAGCCTTTCCAGTCCAACTATGTACGGAACAATGCAAGCCTAGTAGCGGAGGAGGCATACCGTGGTCAAATCTCGTgatgtctatta                                                  >  1:106407/1‑291 (MQ=255)`  
                                                                                                                                                                                                                                                              `ggTAAACAGAAGGAAATGATTGTGCAGTTCCTGACACACGGTATGGAAGGATTGATGACATTCGTAGTACGTACATCATTTCGTGAGGCCATTAAGGACATGCACGAAGAGTATGCAGATAAGGACACTTTCAAACAATCTCCTAGAACAGTACGGGAGGTGTTCTGATGTCCGACTACCTGAAAGTGCTGCAAGCAATCAAAAGTTGCCCTAAGACTTTCAAGACCAAGTATGTACGGAACAATGCGAGCCTCGTAGCAGAGGCCGCTTCCCGTGGTcacaaccctgtctcttatacaca                                                >  1:475043/1‑282 (MQ=255)`  
                                                                                                                                                                                                                                                              `ggTAAACAGAAGGAAATGATTGTCCAGTTCCTGACACACGGTATGGAAGGATTGATGACATTCGTAGTACGTACATCATTTCGTGAGGCCATTAAGGACATGCACGAAGAGTATGCAGATAAGGACTCTTTCAAACAGTCTCCTGCAACAGTACGGGAGGTGTTCTGATGTCTGACTACCTGAAAGTGCTGCAAGCAATCAAAAGTTGCCCTAAGACTTTCCAGTCCATCTATGTACGGAACAATGCGAGCCTCGTAGCGGAGGCCGCTTCCCGTGGTCACATCTCGTGCCTGACTACTAg                                                >  1:288289/1‑301 (MQ=255)`  
                                                                                                                                                                                                                                                              `ggTAAACAGAAGGAAATGATTGTCCAGTTCCTGACACACGGTATGGAAGGATTGATGACATTCGTAGTACGTACATCATTTCGTGAGGCCATTAAGGACATGCACGAAGAGTATGCAGATAAGGACTCTTTCAAACAATCTCCTGCAACAGTACGGGAGGTGTTCTGATGTCTGACTACCTGAAAGTGCTGCAAGCAATCAAAAGTTGCCCTAAGACTTTCCAGTCCAACTATGTACGGAACAATGCGAGCCTCGTAGCGGAGGCCGCTTCCCGTGGTCACATCTCGTGCCTGCCTACTAg                                                >  1:100057/1‑301 (MQ=255)`  
                                                                                                                                                                                                                                                              `aataagagacaGGAAAGGATATTCCAGTTCCTGACACACGGGATGGAAGGATTGATGACATTCGTAGTACGTACATCATTTCGTGAGGCCATTAAGGACATGCACGAAGAGTATGCAGATAAGGACTCTTTCAAACAATCTCCTGCAACAGTACGGGAGGTGTTCTGATGTCTGACTACCTGAAAGTGCTGCAAGCAATCAAAAGTTGCCCTAAGACTTTCCAGTCCAACTATGTACGGAACAATGCGAGCCTCGTAGCGGAGGCCGCTTCCCGTGGTCACATCTCGTGCCTGACTACTAg                                                <  1:520433/291‑1 (MQ=255)`  
                                                                                                                                                                                                                                                               `gTAAACAGAAGGAAATGATTGTCCAGTTCCTGACACACGGTATGGAAGGATTGATGACATTCGTAGTACGTACATCATTTCGTGAGGCCATTAAGGACATGCACGAAGAGTATGCAGATAAGGACTCATTCAAACAATCTCCTGCAACAGTACGGGAGGTGTTCTGATGTCTGACTACCTGAAAGTGCTGCAAGCAATCAAAAGTTGCCCTAAGACTTTCCAGTCCAACTATGTACGGAAAAATGCGAGCCTAGTAGCGGAGGCCGCTTCCCGTGTTCAGATCTCGTGCCTGACTACTAgc                                               >  1:313984/1‑300 (MQ=255)`  
                                                                                                                                                                                                                                                                             `aTGATTGTCCAGTTCCTGACACACGGTATGGAAGGATTGATGACATTCGTAGTACGTACATCATTTCGTGAGGACATTAAGGACATGCACGAAGAGTATGCAGATAAGGACTCTTTCAAACAATCTCCTGCAACAGTACGGGAGGTGTTCTGATGTCTGACTACCTGAAAGTGCTGCAAGCAATCAAAAGTTGCCCTAAGACTTTCCAGTCCAACTATGTACGGAACAATGCGAGCCTCGTAGCGGAGGCCGCTTCCCGTGGTCACATCTCGTGCCTGACTACTAGTGGACGTAACGGTgg                                 <  1:320804/301‑1 (MQ=255)`  
                                                                                                                                                                                                                                                                              `tGATTGTCCAGTTCCTGACACACGGTATGGAAGGATTGATGACATTCGTAGTACGTACATCATTTCGTGAGGCCATTAAGGACATGCACGAAGAGTATGCAGATAAGGACTCTTTCAAACAATCTCCTGCAACAGTACGGGAGGTGTTCTGATGTCTTACTACCTGAAAGTGCTGCAAGCAATCAAAAGTTGCCCTAAGACTTTCCAGTCCAACTATGTACGGAACAATTCGAGCCTCGTAGCGGAGGCCTCTTCCCGTTGTCACCTCGCGTGCCTGCCTACTAGTGGACGTAACgtcggc                                >  1:148980/1‑296 (MQ=255)`  
                                                                                                                                                                                                                                                                                   `gTCCAGTTCCTGACACACGGTATGGAAGGATTGATGACATTCGTAGTACGTACATCATTTCGTGAGGCCATTAAGGACATGCACGAAGAGTATGCAGATAAGGACTCTTTCAAACAATCTCCTGCAACAGTACGGGAGGTGTTATGATGTCTGACTACCTGAAAGTGCTGCAAGCAATCAAAAGTTGCCCTAAGACTTTCCAGTCAAACTATGTACGGAACAATGCAAGCCTCGTAGCGGAGGCCGCTTCCCGTGGTAACATCTCGTGCCTGACTACTAGTGGAGGTAACGGTGGCGCTTg                           >  1:141876/1‑301 (MQ=255)`  
                                                                                                                                                                                                                                                                                      `cAGTTCCTGACACACGGTATGGAAGGATTGATGACATTCGTAGTACGTACATCATTTCGTGAGGCCATTAAGGACATGCACGAAGAGTATGCAGATAAGGACTCTTTCAAACAATCTCCTGCAACAGTACGGGAGGTGTTCTGATGTCTGACTACCTGAAAGTGCTGCAAGCAATCAAAAGTTGCCCTAAGACTTTCCAGTCCAACTATGTACGGAACAATGCGAGCCTCGTAGCGGAGGCCGCTTCCCGTGGTCACATCTCGTGCCTGACTACTAGTGGACGTAACGGTGGCGCTTGGGa                        <  1:33206/301‑1 (MQ=255)`  
                                                                                                                                                                                                                                                                                        `gTTCCTGACACACGGTATGGAAGGATTGATGACATTCGTAGTACGTACATCATTTCGTGAGGCCATTAAGGACATGAACGAAGAGTATGCAGATAAGGACTCTTTCAAACAATCTCCTGCAACAGTACGGGAGGTGTTCTGATGTCTGACTACCTGAAAGTGCTGCAAGCAATCAAAAGTTGCCCTAAGACTTTCCAGTCCAACTATGTACGGAACAATGCGAGCCTCGTAGCGGAGGCCGCTTCCCGTGGTCACATCTCGTGCCTGACTACTAGTGGACGTAACGGTGGCGCTTGGGaaa                      <  1:610836/301‑1 (MQ=255)`  
                                                                                                                                                                                                                                                                                          `tCCTGACACACGGTATGGAAGGATTGATGACATTCGTAGTACGTACATCATTTCGTGAGGCCATTAAGGACATGCACGAAGAGTATGCAGATAAGGACTCTTTCAAACAATCTCCTGCAACAGTACGGGAGGTGTTCTGATGTCTGACTACCTGAAAGTGCTGCAAGCAATCAAACGTTGCCCTAAGACTTTCCAGTCCAACTATGTACGGAACAATGCGAGCCTCGTAGCGGAAGCCGCTTCCCGTGGTCAATGCTCGTGCCTGACTCCTTGTGGACGGAACGGTGGCGTTTGGGAGAtg                    >  1:17855/1‑300 (MQ=255)`  
                                                                                                                                                                                                                                                                                                   `acGGTATGGAAGGATTGATGACATTCGTAGTACGTACATCATTTCGTGAGGCCATTAAGGACATGCACGAAGAGTATGCAGATAAGGACTCTTTCAAACAATCTCCTGCAACAGTACGGGAGGTGTTCTGATGTCTGACTACCTGAAAGTGCTGCAAGCAATCAAAAGTTGCCCTAAGACTTTCCAGTCCAACTATGTACGGAACAATGCGAGCCTCGTAGCGGCGGCCGCTTCCCGTGGTCACATCTCGTGCCTGACTACTAGTGGACGTAACGGTGGCGCTTGGGAAATCACTGCTTcc           >  1:510394/1‑301 (MQ=255)`  
                                                                                                                                                                                                                                                                                                    `cGGTATGGAAGGATTGATGACATTCGTAGTACGTACATCATTTCGTGAGGCCATTAAGGACATGCACGAAGAGTATGCAGATAAGGACTCTTTCAAACAATCTCCTGCAACAGTACGGGAGGTGTTCTGATGTCTGACTACCTGAAAGTGCTGCAAGCAATCAAAAGTTGCCCTAAGACTTTCCAGTCCAACTATGTACGGAACAATGCGAGCCTCGTAGCGGAGGCCGCTTCCCGTGGTCACATCTCGTGCCTGACTACTAGTGGACGTAACGGTGGCGCTTGGGAAATCACTGCTTCCg          >  1:91956/1‑301 (MQ=255)`  
                                                                                                                                                                                                                                                                                                      `gTATGGAAGGATTGATGACATTCGTAGTACGTACATCATTTCGTGAGGCCATTAAGGACATGCACGAAGAGTATGCAGATAAGGACTCTTTCAAACAATCTCCTGCAACAGTCCGGGAGGTGTTCTGATGTCTGACTACCTGAAAGTGCTGCAAGCAATCAAAAGTTGCCCTAAGACTTTCCAGTCCAACTATGTACGGAACAATGCGAGCCTCGTAGCGGAGGCCGCTTCCCGTGGTCACATCTCGTGCCTGACTACTAGTGGACGTAACGGTGGCCCTTGGGAAATCACTGCTTCCGGt        >  1:503706/1‑301 (MQ=255)`  
                                                                                                                                                                                                                                                                                                      `gTATGGAAGGATTGATGACATTCGTAGTACGTACATCATTTCGTGAGGCCATTAAGGACATGCACGAAGAGTATGCAGATAAGGACTCTTTCAAACAATCCCCTGCAACAGTACGGGAGGTGTTCTGATGTCTGACTACCTGAAAGTGCTGCAAGCAATCAAAAGTTGCCCTAAGACTTTCCAGTCCAACTATGTACGGAACAATGCGAGCCTCGTAGCGGAGGCCGCTTCCCGTGGTCACATCTCGTGCCTGACTACTAGTGGACGTAACGGTGGCGCTTGGGAAATCACTGCTTCCGGt        >  1:648683/1‑301 (MQ=255)`  
                                                                                                                                                                                                                                                                                                      `gTATGGAAGGACTGATGACATTCGTAGTACGTACATCATTTCGTGAGGCCATTAAGGACATGCACGAAGAGTATGCAGATAAGGACTCTTTCAAACAATCTCCTGCAACAGTACGGGAGGTGTTCTGATGTCTGACTACCTGAAAGTGCTGCAAGCAATCAAAAGTTGCCCTAAGACTTTCCAGTCCAACTATGTACGGAACAATGCGAGCCTCGTAGCGGAGGCCGCTTCCCGTGGTCACATCTCGTGCCTGACTACTAGTGGACGTAACGGTGGCGCTTGGGAAATCACTGCTTCCTGt        >  1:437664/1‑301 (MQ=255)`  
                                                                                                                                                                                                                                                                                                        `aTGGAAGGATTGATGACATTCGTAGTACGTACATCATTTCGTGAGGCCATTAAGGACATGCACGAAGAGTATGCAGATAAGGACTCTTTCAAACAATCTCCTGCAACAGTACGGGAGGTGTTCTGATGTCTGACTACCTGAAAGTGCTGCAAGCAATCAAAAGTTGCCCTAAGACTTTCCAGTCCAACTATGTACGGAACAATGCGAGCCTCGTAGCGGAGGCCGCTTCCCGTGGTCACATCTCGTGCCTGACTACTAGTGGACGTAACGGTGGCGCTTGGGAAATCACTGCTTCCGGTAc      >  1:581117/1‑301 (MQ=255)`  

                                                                                                                                                                                                                                                                                                           `|`                                                                                                                                                                                                                                                                                                               
`GACATTCTTGCGGTTGTGGTTGACGAAGATGTGCTTATTTTCACATGGGACGAAGTAAAAGGTAAGACATCCATGTGTGTCGGCAAGAGAAACAAAGGCATAAAACTATAGGAGAAATTATTATGGCTATGACAAAGAAATTTAAAGTGTCCTTCGACGTTACCGCAAAGATGTCGTCTGACGTTCAGGCAATCTTAGAGAAAGATATGCTGCATCTATGTAAGCAGGTCGGCTCAGGTGCGATTGTCCCCAATGGTAAACAGAAGGAAATGATTGTCCAGTTCCTGACACACGGTATGGAAGGATTGATGACATTCGTAGTACGTACATCATTTCGTGAGGCCATTAAGGACATGCACGAAGAGTATGCAGATAAGGACTCTTTCAAACAATCTCCTGCAACAGTACGGGAGGTGTTCTGATGTCTGACTACCTGAAAGTGCTGCAAGCAATCAAAAGTTGCCCTAAGACTTTCCAGTCCAACTATGTACGGAACAATGCGAGCCTCGTAGCGGAGGCCGCTTCCCGTGGTCACATCTCGTGCCTGACTACTAGTGGACGTAACGGTGGCGCTTGGGAAATCACTGCTTCCGGTACTCGC  >  NC_001604.1/16729‑17329`

  

Alignment Legend

Aligned base mismatch/match (shaded by quality score): `ATCG/ATCG` `< 3 ≤` `ATCG/ATCG` `< 10 ≤` `ATCG/ATCG` `< 21 ≤` `ATCG/ATCG` `< 34 ≤` `ATCG/ATCG` `< 38 ≤` `ATCG/ATCG`

Unaligned base: `atcg`    Masked matching base: `atcg`    Alignment gap: `‑`    Deleted base: `‑`

Reads not counted as support for junction

read\_name Not counted due to insufficient overlap past the breakpoint.

read\_name Not counted due to not crossing MOB target site duplication.
